# Imaging-Guided Treatment for Cardiac Amyloidosis

**DOI:** 10.1007/s11886-022-01703-7

**Published:** 2022-05-07

**Authors:** Adam Ioannou, Rishi Patel, Julian D. Gillmore, Marianna Fontana

**Affiliations:** National Amyloidosis Centre, University College London, Royal Free Campus, Rowland Hill Street, London, NW3 2PF UK

**Keywords:** Cardiac amyloidosis, Echocardiography, Cardiac magnetic resonance imaging, Immunoglobulin light chains, Transthyretin

## Abstract

**Purpose of Review:**

This review will explore the role of cardiac imaging in guiding treatment in the two most commonly encountered subtypes of cardiac amyloidosis (immunoglobulin light-chain amyloidosis [AL] and transthyretin amyloidosis [ATTR]).

**Recent Findings:**

Advances in multi-parametric cardiac imaging involving a combination of bone scintigraphy, echocardiography and cardiac magnetic resonance imaging have resulted in earlier diagnosis and initiation of treatment, while the evolution of techniques such as longitudinal strain and extracellular volume quantification allow clinicians to track individuals’ response to treatment. Imaging developments have led to a deeper understanding of the disease process and treatment mechanisms, which in combination result in improved patient outcomes.

**Summary:**

The rapidly expanding treatment regimens for cardiac amyloidosis have led to an even greater reliance on cardiac imaging to help establish an accurate diagnosis, monitor treatment response and aid the adjustment of treatment strategies accordingly.

## Introduction

Systemic amyloidosis describes a heterogenous group of diseases characterised by deposition of amyloid fibrils within the extracellular space of various organs. Amyloid fibrils are formed when normally soluble precursor proteins misfold into insoluble, beta-pleated sheets, which are resistant to proteolysis and histologically identifiable by apple-green birefringence when stained with Congo-Red dye and examined under cross-polarised light. Disease occurs when aggregation of amyloid fibrils is sufficient to disrupt the structure, integrity and function of the affected organ [1, 2].

Cardiac amyloidosis (CA) occurs when amyloid fibrils deposit within the myocardial extracellular space, causing interruption and distortion of myocardial contractile elements, impaired ventricular relaxation, and subsequent systolic and diastolic dysfunction. Although amyloidosis is a multi-system disease, cardiac involvement remains the leading cause of mortality [3, 4]. Over 30 different human precursor proteins can form amyloid fibrils [1, 2], but the overwhelming majority of CA cases result from misfolded immunoglobulin light-chain (light-chain amyloidosis [AL]) and transthyretin (transthyretin amyloidosis [ATTR]) proteins [5, 6].

AL amyloidosis is a rare condition [5, 7] and occurs as the result of deposition of misfolded immunoglobulin light chains, produced by an abnormal clonal proliferation of plasma cells [5, 7]. Cardiac involvement is present in up to 70% of cases [1, 3] and historically was associated with a poor prognosis, with a median survival of 6 months if left untreated [8]. Advances in chemotherapeutic regimens that target clonal plasma cells and supress AL-amyloid production have substantially improved survival [9, 10]. If successful, novel treatment agents combined with autologous stem cell transplantation can result in median survival exceeding 4 years [11].

ATTR amyloidosis is caused by misfolding of the transthyretin protein. This process can be secondary to inherited genetic mutations, known as hereditary ATTR (hATTR); or an acquired process associated with ageing, known as wild-type ATTR (wtATTR) [12]. The heart is the main major organ involved [13, 14], while extra-cardiac sites of infiltration cause carpal tunnel syndrome, lumbar spine stenosis and tendinopathies [15–17]. Disease progression occurs at a slower rate than AL amyloidosis, resulting in a median survival of 3–5 years without treatment [18]. Although the diagnosis of wtATTR in patients presenting with heart failure with preserved ejection fraction (HFpEF) has increased in recent years [13, 19], wtATTR still remains an under-recognised cause of HF [20]. hATTR presents at a younger age, with a mixed phenotype comprising of neuropathy and/or restrictive cardiomyopathy [21]. Over 130-pathogenic mutations are responsible for hATTR, but only a small handful are implicated in the majority of hATTR cases [22–24].

CA is a heterogenous complex disease process, with clinical presentation and subsequent treatment strategies varying, depending on the underlying amyloid type and disease severity. Improved understanding of each individual patient’s cardiac phenotype through multi-modality cardiac imaging facilitates a comprehensive evaluation of treatment response, which has important implications on clinical care and prognosis. This review will evaluate the role of cardiac imaging in the treatment response.

## Overview of Imaging Modalities Used in Cardiac Amyloidosis

### Echocardiography

Echocardiography is widely available, relatively inexpensive and often the first-line investigation for patients with HF symptoms. CA is characterised by biventricular wall thickening, with symmetrical left ventricular (LV) wall thickening more classically seen in AL amyloidosis and asymmetrical septal thickening in ATTR amyloidosis [25, 26]. Stiffening of the myocardium results in impaired relaxation, raised filling pressures and diastolic dysfunction, which is reflected in an increased E/e’. Raised filling pressures coupled with atrial amyloid infiltration lead to atrial dilation and dysfunction with a proportion of patients showing atrial electromechanical dissociation [27, 28]. Although CA has been classically regarded as a cause of HFpEF, this terminology underestimates the effect of amyloid infiltration on systolic function. Longitudinal function is typically affected before radial contraction and was classically reflected in a reduced mitral and tricuspid annular plane systolic excursion (MAPSE and TAPSE) [29, 30]. Advances in longitudinal strain (LS) measurements through speckle tracking provide more accurate estimations of longitudinal function, and regional strain measurements that predominantly affect the basal segments with apical sparing. These characteristic features produce the typical ‘bull’s eye pattern’ and are sensitive (92%) and specific (82%) in discriminating CA from LV hypertrophy. Mild forms of apical sparing have been described in a minority of patients with HFpEF who do not have CA, but the overwhelming majority of patients with this strain pattern are subsequently diagnosed with CA. Despite good diagnostic accuracy, speckle tracking does have some limitations. It relies heavily on image quality and good endocardial definition, which can be highly variable between patients. In those with poor image quality, the accuracy of speckle tracking is reduced, and hence it becomes a less reliable diagnostic tool. The reliance on manual contouring results in inter-operator variability and reduced precision, which should be considered when tracking disease progression, and tracking treatment response with serial strain measurements [31, 32]. Amyloid valvular infiltration can also present with valvular thickening and regurgitation. Although these findings are not sensitive or specific, moderate-to-severe valvular insufficiency is a significant predictor of prognosis [27]. In addition, pericardial and pleural effusions are not uncommon findings [4].

### Cardiac Magnetic Resonance Imaging

Cardiac magnetic resonance (CMR) imaging allows detailed tissue characterisation that provides accurate information on myocardial composition and can differentiate between CA and other cardiomyopathies. The extracellular matrix expansion caused by amyloid fibril deposition can be well visualised with administration of gadolinium-based contrast agents, which accumulates in the extracellular space. Resultant patterns of late gadolinium enhancement (LGE) are characteristically circumferential, diffuse and progresses from subendocardial to transmural enhancement [33]. LGE can visualise the continuum of cardiac amyloid infiltration [34], while the degree of transmurality can accurately predict prognosis [35]. The main drawback of LGE is that it is not a quantitative measurement, which makes it difficult to track changes over time; and gadolinium use is relatively contraindicated in patients with chronic kidney disease (estimated glomerular filtration rate < 30 ml/min/1.73 m^2^), due to a potential risk of nephrogenic systemic fibrosis. This is a particular issue in CA, as many patients (especially those with AL amyloidosis) have concomitant renal impairment.

These limitations can be overcome by T1 mapping, which gives a quantitative pixel-based measure of myocardial longitudinal relaxation time. Elevated native T1 (pre-contrast) is a sensitive marker of early amyloid infiltration, and may even be elevated before LGE development [36–38]. Gadolinium contrast administration in conjunction with pre and post T1 mapping enables the estimation of myocardial extracellular volume (ECV) from the ratio of signal change in blood and myocardium after contrast. Due to the pathophysiological ECV expansion that occurs, elevated ECV measurements are highly sensitive and specific for diagnosing CA [39, 40]. ECV demonstrates higher diagnostic accuracy than LGE, correlates well with other biomarkers of amyloid disease burden and prognosis and, most importantly, can be used to measure the continuum of CA infiltration, from early CA involvement to severe degree of CA burden. Serial ECV measurements can track disease progression and treatment response. ECV measurements are corrected by the haematocrit level, but there is the potential that large changes in haematocrit could affect ECV measurements, and influence the ability to track changes over time. ECV is a surrogate marker for amyloid burden and is not a direct measure of amyloid load; however, despite these pitfalls, it is the best available method of tracking changes over time [41]. Routine CMR imaging has demonstrated a high diagnostic performance in hepatic and splenic ECV mapping. These measurements allow identification and quantification of extra-cardiac amyloid burden, without requiring any additional imaging sequences and may provide a means to track the change in hepatic and splenic amyloid load in response to treatment [42]. T2 mapping serves as a surrogate marker of myocardial oedema, by estimating the myocardial water content. Classically it has been used in the diagnosis of inflammatory conditions such as myocarditis, but more recently has been utilised in the assessment of CA, with elevated T2 values being associated with mortality, while AL-amyloidosis patients undergoing active treatment have significantly lower values than those untreated. Therefore, T2 mapping could potentially track disease progression and treatment response [43].

### Bone Scintigraphy

Bone scintigraphy was first repurposed in the 1980s, when an incidental finding of increased cardiac uptake of 99mTc-phosphate derivatives was observed in CA. To date, the underlying mechanism behind localisation of these agents to CA remains poorly understood. In 2005, a seminal study demonstrated the diagnostic potential of ^99m^Technetium-labelled 3,3-dicarboxypropane-2, 1-diphosphonate (^99m^Tc-DPD) in identifying ATTR-CA [44]. Recent studies confirmed the high sensitivity of ^99m^Tc-DPD cardiac uptake in diagnosing ATTR-CA [45], but of note, only 40% of patients with AL-CA have any cardiac uptake, and therefore concomitant screening for excessive immunoglobulin production remains necessary. The impressive sensitivity of ^99m^Tc-DPD has been utilised in developing a non-biopsy diagnostic algorithm for ATTR-CA. If CA is clinically suspected based on echocardiography or CMR, and plasma cell dyscrasia has been excluded (utilising serum free light chain (FLC) with serum/urine immunofixation electrophoresis), then ^99m^Tc-DPD grade 2–3 uptake is confirmatory of ATTR-CA. This imaging-based algorithm demonstrated a high specificity and positive predictive value (both 98%) in a large multi-centre trial, and has since negated the need for biopsy in a multitude of patients, and assisted the timely initiation of treatment [46]. Data on monitoring disease activity through serial scans is limited. Once uptake is established, visual changes are unlikely to occur; therefore, ^99m^Tc-DPD-scintigraphy is primarily a diagnostic tool, rather than used to track treatment response.

### Single-Photon Emission Computed Tomography

Single-photon emission computed tomography (SPECT) can add a three-dimensional visualisation to planar bone scintigraphy. ^99m^Tc-DPD tracer injection followed by SPECT and non-contrast CT allows more detailed and accurate assessment of radiotracer uptake within the myocardium [47–49]. It provides a quantitative measure of radiotracer uptake that correlates well with serum cardiac biomarkers and echocardiographic strain measurements [49]. Although this adds valuable diagnostic information, like planar bone scintigraphy, it remains a diagnostic tool rather than an investigation that can accurately track treatment response.

### Positron Emission Tomography

Positron emission tomography (PET) was identified as another form of cardiac imaging with diagnostic potential. Several PET tracers, such as 18F-florbetapir, 18F-florbetaben, 18F-flutemetamol and 11C-Pittsburgh B (11C-PiB), have been successfully used to diagnose CA [50–56]. These tracers bind to amyloid fibrils and allow quantitative measurement of amyloid burden. Small studies have demonstrated increased tracer uptake in CA compared to controls [50–52], and a recent meta-analysis concluded that PET has a high sensitivity and specificity for detecting CA [57]. Although PET has demonstrated utility in diagnosis, at present there is a lack of robust data to support its use in tracking treatment response.

## Cardiac Imaging and Tracking Treatment Response in ATTR Cardiac Amyloidosis

Current therapeutic strategies aim at reducing the deposition of ATTR in the myocardium. This is achieved either through stabilisation of the transthyretin tetramer to prevent dissociation into pathological monomers and oligomers that form amyloid fibrils [58] or by reducing hepatic synthesis of ATTR by disrupting the relevant messenger RNA (mRNA) [59]. Early diagnosis and initiation of treatment are associated with improved outcomes. With a constantly expanding armamentarium of treatments, it is essential that early identification of treatment responders can be distinguished from non-responders, which may facilitate early alteration in their treatment regimen [3, 4].

### Diflunisal

Diflunisal is a non-steroidal anti-inflammatory drug used as a transthyretin stabiliser, and was initially used in patients with hATTR polyneuropathy [60], but has since been studied in CA. A retrospective analysis demonstrated those on diflunisal had a reduction in mortality or orthoptic heart transplant compared to those not on a TTR stabiliser [61]. A prospective study showed 34 patients treated with diflunisal had a significant improvement in apical LV rotation/torsion, without a deterioration in longitudinal and radial strain at 1 year [62]. A recent retrospective study of 81 patients confirmed that diflunisal stabilised global longitudinal strain (GLS), while it deteriorated in the untreated group [63].

### Tafamidis

Tafamidis is another transthyretin binder that stabilises the tetramer and prevents its dissociation. To date, it is the only medication licenced purely for treatment of ATTR-CA, and all other disease modifiers are only licenced for the treatment of ATTR polyneuropathy. In a multi-centre trial involving 441 patients, tafamidis reduced all-cause mortality, and functional decline when compared to placebo. This was reflected in an attenuated decrease in stroke volume following 30 months of treatment [58]. While there have been no large-scale studies to assess change in CMR parameters during treatment, a recent case report demonstrated that tafamidis treatment resulted in stabilisation of CA, with measurements of LV mass, native T1 and ECV all remaining stable after 1 year of treatment. Despite being promising, these findings would need to be validated in a prospective trial [64].

### Patisiran

Patisiran is an RNA interference therapeutic that acts as a TTR-gene silencer, by disrupting the transthyretin mRNA, hence reducing its hepatic synthesis. It is currently only licenced for treatment of ATTR polyneuropathy, after the large-scale APOLLO study demonstrated that treatment improved multiple neuropathic manifestations of ATTR amyloidosis [59]. Post hoc analysis of the 126 patients with CA (who underwent transthoracic echocardiography) showed that patisiran reduced mean LV wall thickness, relative wall thickness, GLS and increased cardiac output after 18 months of treatment [65]. A study of 16 patients treated with patisiran in combination with diflunisal demonstrated a reduction in CMR-derived ECV alongside a reduction in serum TTR and cardiac biomarkers. Interestingly, 15 patients had a reduction in ^99m^Tc-DPD cardiac uptake after 12 months of therapy. This was typically accompanied by a reduction in skeletal muscle and soft tissue uptake, and a corresponding increase in bone signal. However, the dynamics and kinetics of ^99m^Tc-DPD binding to the bones, soft tissues and myocardium in ATTR-CA may fluctuate; hence, a change in uptake in any of these compartments will affect the appearance of cardiac uptake. The authors advised caution in interpreting such images, and ascribing observed changes exclusively to reduced CA burden in the absence of biochemical, echocardiographic or CMR evidence of disease regression. If these encouraging results are reproducible on a larger scale, it would represent a direct measure of CA regression in response to treatment [66••].

### Inotersen

Inotersen is an antisense oligodeoxynucleotide that similarly degrades TTR mRNA. It has been successfully trialled in hATTR polyneuropathy and since been licenced for this indication. A small subgroup analysis demonstrated preservation of LV wall thickness and mass on CMR, but this was not compared to the placebo group [67]. These findings were supported by a small study of 8 patients who showed stabilisation of LV wall thickness, LV mass and GLS after 1 year of treatment [68], while a prospective cohort study of 33 patients with ATTR-CA demonstrated that inotersen treatment actually led to a reduction in LV mass on CMR after 2 years of treatment [69]. CMR can measure changes in tissue characterisation that occur during disease regression, ahead of observed improvements in conventional structural and functional echocardiographic parameters; but as with patisiran, a large-scale trial is needed to confirm the ability of inotersen to induce disease regression.

### Novel Therapies in Development

As the understanding of the underlying pathophysiology responsible for ATTR-CA improves, the number of potential treatment targets and therapeutic options increases. The ever-expanding treatment options include various novel therapies that are currently being trialled. Acoramidis (TTR stabiliser) [70] and vutrisiran (RNA interface therapeutic) [71] are currently in phase-3 trials, while PRX004 (monoclonal antibody against ATTR) is in a phase-1 trial [72]. NTLA-2001 (genome-editing drug targeting the TTR gene) has been safely administered in a cohort of hATTR-polyneuropathy patients, in whom it induced TTR knockout, with a significant reduction in serum TTR concentrations. NTLA-2001 is currently being trailed in patients with ATTR-CA, and if successful has the potential to revolutionise ATTR-CA treatment [73, 74]. Serial echocardiograms and CMR scans will prove valuable in determining whether these therapies can halt disease progression, or even induce regression.

## Cardiac Imaging and Tracking Treatment Response in AL Cardiac Amyloidosis

The mainstay of treatment in AL-CA is cytotoxic chemotherapy, aimed at suppressing monoclonal immunoglobulin light-chain production, halting ongoing amyloid deposition and allowing gradual organ recovery [3, 5]. A rapid and deep haematological response is associated with improved survival and outcomes even in advanced disease. In recent years, improvements in survival have been further enhanced by the substantial improvements in efficacy and toxicity of chemotherapeutic regimens [9, 10]. However, the benefits must be balanced against risks of sinister side effects, which can be difficult in those with advanced disease and multi-organ dysfunction. Stem cell transplantation is reserved for those with a good functional baseline, who have not developed advanced cardiac or renal failure [11]. Novel therapies include daratumumab (monoclonal antibody against CD38), which is currently being assessed in a phase-2 trial, and may offer an exciting alternative for those deemed at a high risk of chemotherapy-related adverse events [75]. In view of not only the substantial reduction in mortality associated with a response to treatment, but also the potential for severe side effects, it is essential to determine whether a patient’s disease has remained stable, progressed or regressed.

### Monitoring the Response to Chemotherapy with Echocardiography

**Fig. 1:**
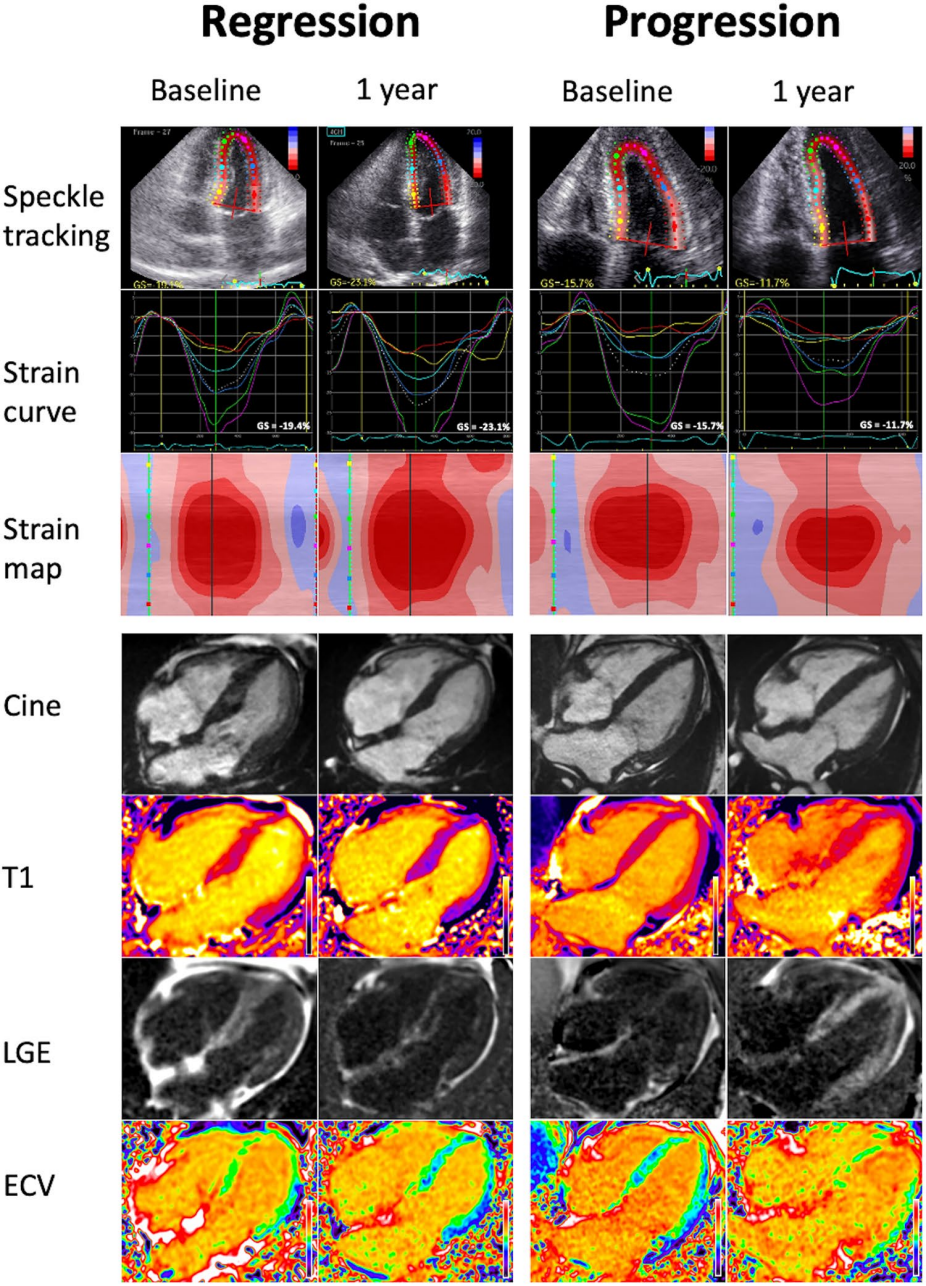
Top panel: echocardiographic global strain (GS) measurements demonstrating an improvement in a patient with a good haematological response to chemotherapy, and a deterioration in another patient with a poor haematological response. Bottom panel: cardiac magnetic resonance imaging demonstrating a reduction in native T1, late gadolinium enhancement (LGE) and extracellular volume (ECV) in a patient with a good haematological response to chemotherapy, and a deterioration in another patient with a poor haematological response

The response to chemotherapy has traditionally been classified by assessing the reduction in FLC, and this classification has subsequently been used to assess whether a haematological response correlates with stabilisation or even regression of CA. An early study of 41 patients, which defined haematological response as a reduction in FLC > 50%, demonstrated that haematological response had a weak correlation with reduced E/e’ and left atrial stiffness, but did not result in GLS improving [76]. In contrast, a retrospective analysis of 61 patients, which separated patients into complete responders (normal FLC ratio and negative serum/urine immunofixation) and non-complete responders, demonstrated that complete responders had a significant improvement in LS, and this correlated with reduced brain natriuretic peptide (BNP) and troponin-I. Interestingly there were no changes observed in wall thickness, ejection fraction (EF) or diastolic function [77]. These findings are supported by a recent study of 915 patients, which concluded those achieving a complete haematological response had improved LS, compared to those who did not, and was associated with reduced N-terminal pro-BNP and all-cause mortality (Fig. 1) [78••]. Based on these findings, it appears a complete haematological response, rather than a partial response is required to improve LS. Even a small residual amount of AL amyloid is likely to exert pathological cardiotoxicity and prevent cardiac recovery. Improved LS acts as a surrogate marker for a good treatment response, and reduced mortality.

### Monitoring the Response to Stem Cell Transplantation with Echocardiography

High-dose melphalan-based chemotherapy has been combined with stem cell transplantation to successfully improve prognosis. A retrospective analysis of 55 patients with a complete haematological response demonstrated a significant reduction in wall thickness, while in partial responders there was a trend for wall thickness to increase [79]. A subsequent study of 187 patients undergoing the same treatment demonstrated those who achieved a haematological response were significantly more likely to see favourable changes in cardiac structure and function. A cardiac response, defined as a reduction in interventricular septal wall thickness ≥ 2 mm and improvement in EF ≥ 20%, was observed in 41% of patients undergoing treatment, and was associated with reduced all-cause mortality [80]. In contrast, a small study of 82 patients undergoing autologous stem cell transplant demonstrated that at 1 year, 59% had achieved a complete haematological response, of which only 61% (27 patients) had a follow-up echocardiogram that showed no significant change in wall thickness, systolic or diastolic function [81]. However, the small cohort of patients and substantial number who did not have a follow-up echocardiogram may explain why no significant difference was observed. In recent analysis of 72 patients (38 retrospective and 34 prospective) undergoing chemotherapy (29% of whom had a stem cell transplantation), those with adverse events had a significant decline in GLS and increase in E/e’, compared with those classified as having event-free survival [82]. GLS is a strong predictor of survival in AL-CA, and therefore it is unsurprising that in patients whereby treatment can attenuate a pathological decline in GLS, or even drive improvements, this will result in reduced morbidity and mortality [76].

### Monitoring the Response to Chemotherapy with Cardiac Magnetic Resonance Imaging

A prospective study of 65 patients with confirmed AL-CA sought to determine the difference in CMR phenotype in those undergoing chemotherapy treatment compared to those left untreated. Those treated had a significantly lower myocardial T2 and a trend for a lower ECV compared to those left untreated. The lower ECV was notably not significant and the difference observed could be in part due to reduced myocardial oedema, rather than just a lower CA burden [43].

A recent retrospective analysis sought to evaluate the use of serial CMR scans in monitoring treatment response in 31 patients with AL-CA. A complete haematological response was achieved in 36%, and a very good response (defined as FLC < 40 mg/dl) in 29%. A significant reduction in ECV attaining the CMR criteria for CA regression occurred in 13 patients, and a complete or very good response was associated with a reduction in ECV (92%). The reduction in ECV led to a reduced LV mass in 7 (54%) patients who regressed, and was associated with an improved LGE pattern in 5 (38%) patients. This demonstrates that cardiac regression occurs in a significant number of patients who respond to treatment. The reversal of LGE seen in those with a reduction in ECV is compelling evidence that this reduction is not just due to a reduction in myocardial oedema, but also CA regression (Fig. 1) [83•].

Up until now, the CA response to treatment has been inferred from parameters measured on echocardiography, with varying degrees of success. LS appears to be the most sensitive echocardiographic marker of treatment response, with improvements being seen in the largest study of patients undergoing treatment [78••], but smaller studies have yielded mixed results [76]. To date, only one small study has shown a reduction in wall thickness on echocardiography [80], while the majority have not demonstrated a significant change. The increased resolution and deep tissue characterisation obtained through CMR may allow a more accurate quantification of the CA burden. The ability to track these changes has the potential to redefine the cardiac response to treatment, and enable stratification of patients with a lower risk of progression and better prognosis, in whom the need to intensify chemotherapy may not be required.

## Future Perspectives

The ever-expanding and evolving treatment options for CA mean clinician’s reliance on cardiac imaging for early diagnosis, accurate prognostication and tracking treatment response will increase exponentially. Early diagnosis, through a multi-parametric imaging approach (advantages and limitations of which are outlined in Table 1), leads to prompt initiation of disease-modifying therapy and improved outcomes [84]. A greater awareness of CA amongst clinicians and advancements in cardiac imaging has resulted in patients being diagnosed at an earlier disease stage [85]. Genetic screening combined with cardiac imaging can result in asymptomatic patients being diagnosed with ATTR-CA, before any structural changes are detectable on echocardiography, just on the basis of mild ^99m^Tc-DPD cardiac uptake. More data are needed to guide decisions on in whom and when to initiate treatment. Further studies are required to determine whether treatments should be initiated at very early stages of disease, and which treatments should be used at each disease stage.

**Table 1:** Comparisons of the benefits and limitations of transthoracic echocardiography, cardiac magnetic resonance imaging and bone scintigraphy

	**Transthoracic echocardiogram**	**Cardiac magnetic resonance imaging**	**Bone scintigraphy**
Availability	Widely available in most secondary care settings	Only available at tertiary centres	Only available at tertiary centres
Cost estimates based on UK NHS tariffs (2020/2021)	£58	£586	£198
Clinical information	Provides a detailed assessment of the systolic and diastolic cardiac function, and valvular function. Does not provide any tissue characterisation	Provides detailed tissue characterisation of the myocardium and assessment of systolic function. Allows other myocardial diseases to be identified. Limited information on valvular function and no information on diastology	Provides qualitative information on amyloid infiltration, but does not provide data on cardiac structure or function
Diagnostic utility	Characteristic features of biventricular thickening, diastolic dysfunction and impaired longitudinal function occur with a moderate disease burden. Early amyloid infiltration is often missed	Characteristic elevated native T1, LGE patterns and elevated ECV measurements are typical of CA and allow early amyloid infiltration to be detected	Very high sensitivity for ATTR-CA, and moderate sensitivity for ATTR-CA. Very high specificity for AL and ATTR-CA
Ability to track treatment response	Improvements in LS can indicate regression in AL-CA. Static LS measurements indicate disease stabilisation in ATTR	Reduction in ECV measurements can indicate disease regression in AL and ATTR-CA	Unable to track treatment response
Time required	20–30 min	40–60 min	Scan takes place 2 h post tracer injection and takes 30 min
Practical benefits	- Portable - Safe in pregnancy - Non-invasive - No exposure to ionising radiation	- Image quality not affected by body habitus - No exposure to ionising radiation	- Unaffected by body habitus - Not operator dependant
Practical limitations	- Image quality is affected by body habitus and concurrent pulmonary pathology - Highly operator dependant	- Image quality is affected by breathing and arrhythmias - Requires gadolinium contrast (relatively contraindicated in chronic kidney disease) - Absolutely contraindicated in patients with non-MRI safe metalwork - Not safe in pregnancy	- Not safe in pregnancy - Not safe in breastfeeding - Involves exposure to ionising radiation

*AL* immunoglobulin light-chain amyloidosis, *ATTR* transthyretin amyloidosis, *CA* cardiac amyloidosis, *ECV* extracellular volume, *LGE* late gadolinium enhancement, *LS* longitudinal strain, *MRI* magnetic resonance imaging, *NHS* National Health Service, *UK* United Kingdom

Early studies relied on echocardiography to aid diagnostics and track treatment response; however, more recent studies have successfully demonstrated the utility of CMR. The increased resolution and novel methods of myocardial tissue characterisation have already been successful in tracking treatment response. CMR has demonstrated that novel therapies not only can halt disease progression, but also can importantly induce disease regression [66••, 83•]. These findings will spur the expansion of CMR use in tracking treatment response. The main barricade preventing this expansion is cost. CMR is regarded as a specialised form of imaging, only available in tertiary centres. The centralisation of healthcare services, with all patients requiring investigation being managed in specialist centres, may overcome this barrier, but relies on physicians in non-specialist centres having a low index of suspicion for CA and low threshold to refer patients. Therefore, we would suggest that all patients with a suspicion of CA should be referred to tertiary centres for further investigation, even if the suspicion is low or the diagnosis is suspected to be very early in the disease process.

Although echocardiography is less sensitive, it remains the most widely available and inexpensive form of cardiac imaging. Improvements in sensitivity could play a central role in guiding treatment, something which may be assisted with the emergence of data science and its application for echocardiographic analysis. The utility of artificial intelligence (AI) and more specifically convolutional neural networks is likely to revolutionise all areas of medicine, and has already shown great promise in cardiovascular imaging [86–88]. The elimination of manual operator contouring allows AI to provide accurate and robust measurements of structural and functional parameters, including EF and strain-derived parameters, with a significant improvement in precision. These advantages mean automated measurements correlate far better with patient outcomes than manual measurements [89].

Early diagnosis of CA facilitated through AI-driven image analysis would unequivocally impact prognosis, with studies in CA showing better outcomes amongst patients treated with disease-modifying therapies, early in their disease [3, 4, 84]. This technology may uncover additional echocardiographic parameters that can track treatment response. There is an unmet need for early identification of patients who are responding poorly to treatment to allow alternative treatments to be administered. The ability to recognise responders and non-responders is likely to inform development of individually tailored treatments in the future. The application of AI to analysis of echocardiograms could revolutionise the diagnostic potential of each scan, uncover those with CA who would otherwise remain undiagnosed and help clinicians correctly characterise patients, stratify their disease and track treatment response. Despite the amazing potential of such a novel application, there is still a long way to go in making this aspiration come to fruition.

## Conclusion

Advances in imaging techniques have transformed the treatment landscape of CA. The enhanced sensitivity of multi-parametric imaging has led to earlier diagnosis, treatment initiation and subsequent improved outcomes. Breakthroughs in modern imaging techniques such as strain-derived measurements in echocardiography and ECV quantification in CMR have helped clinicians understand how patients respond to treatment and allowed clinicians to tailor treatment strategies to each individual. The evolution of cardiac imaging is a key component in the treatment pathway for CA. Each development results in a greater understanding of the disease process and in turn helps guide treatment strategies.

## References

[1] Wechalekar AD, Gillmore JD, Hawkins PN (2016). Systemic amyloidosis. Lancet.

[2] Lachmann HJ, Hawkins PN (2006). Systemic amyloidosis. Curr Opin Pharmacol.

[3] Martinez-Naharro A, Hawkins PN, Fontana M (2018). Cardiac amyloidosis Clin Med (Lond).

[4] Chacko L, Martone R, Cappelli F, Fontana M. Cardiac amyloidosis: updates in imaging. Curr Cardiol Rep; 2019;21. 10.1007/s11886-019-1180-2 PMC6677705 31375984

[5] Falk RH, Alexander KM, Liao R, Dorbala S (2016). AL (light-chain) cardiac amyloidosis: a review of diagnosis and therapy. J Am Coll Cardiol.

[6] Fontana M, Banypersad SM, Treibel TA, Abdel-Gadir A, Maestrini V, Lane T (2015). Differential myocyte responses in patients with cardiac transthyretin amyloidosis and light-chain amyloidosis: a cardiac MR imaging study. Radiology.

[7] Quock TP, Yan T, Chang E, Guthrie S, Broder MS (2018). Epidemiology of AL amyloidosis: a real-world study using US claims data. Blood Adv.

[8] Kyle RA, Linos A, Beard CM, Linke RP, Gertz MA, O’Fallon WM (1992). Incidence and natural history of primary systemic amyloidosis in Olmsted County, Minnesota, 1950 Through 1989. Blood.

[9] Kastritis E, Leleu X, Arnulf B, Zamagni E, Cibeira MT, Kwok F (2020). Bortezomib, melphalan, and dexamethasone for light-chain amyloidosis. J Clin Oncol.

[10] Palladini G, Sachchithanantham S, Milani P, Gillmore J, Foli A, Lachmann H (2015). A European collaborative study of cyclophosphamide, bortezomib, and dexamethasone in upfront treatment of systemic AL amyloidosis. Blood.

[11] Madan S, Kumar SK, Dispenzieri A, Lacy MQ, Hayman SR, Buadi FK, et al. High-dose melphalan and peripheral blood stem cell transplantation for light-chain amyloidosis with cardiac involvement. Am J Hematol 2012;119:1117. 10.1182/blood-2011-07-370031 PMC3277349 22147893

[12] Sipe JD, Benson MD, Buxbaum JN, Ikeda SI, Merlini G, Saraiva MJM (2016). Amyloid fibril proteins and amyloidosis: chemical identification and clinical classification International Society of Amyloidosis 2016 Nomenclature Guidelines. Amyloid.

[13] González-López E, Gallego-Delgado M, Guzzo-Merello G, de Haro-Del Moral FJ, Cobo-Marcos M, Robles C (2015). Wild-type transthyretin amyloidosis as a cause of heart failure with preserved ejection fraction. Eur Heart J.

[14] Pinney JH, Whelan CJ, Petrie A, Dungu J, Banypersad SM, Sattianayagam P, et al. Senile systemic amyloidosis: clinical features at presentation and outcome. J Am Heart Assoc 2013;2. 10.1161/JAHA.113.000098 PMC3647259 23608605

[15] Carr AS, Pelayo-Negro AL, Evans MRB, Laurà M, Blake J, Stancanelli C (2016). A study of the neuropathy associated with transthyretin amyloidosis (ATTR) in the UK. J Neurol Neurosurg Psychiatry.

[16] Yanagisawa A, Ueda M, Sueyoshi T, Okada T, Fujimoto T, Ogi Y (2015). Amyloid deposits derived from transthyretin in the ligamentum flavum as related to lumbar spinal canal stenosis. Mod Pathol.

[17] Geller HI, Singh A, Alexander KM, Mirto TM, Falk RH (2017). Association between ruptured distal biceps tendon and wild-type transthyretin cardiac amyloidosis. JAMA.

[18] Arruda-Olson AM, Zeldenrust SR, Dispenzieri A, Gertz MA, Miller FA, Bielinski SJ (2013). Genotype, echocardiography, and survival in familial transthyretin amyloidosis. Amyloid.

[19] Castano A, Narotsky DL, Hamid N, Khalique OK, Morgenstern R, DeLuca A (2017). Unveiling transthyretin cardiac amyloidosis and its predictors among elderly patients with severe aortic stenosis undergoing transcatheter aortic valve replacement. Eur Heart J.

[20] Tanskanen M, Peuralinna T, Polvikoski T, Notkola IL, Sulkava R, Hardy J (2008). Senile systemic amyloidosis affects 25% of the very aged and associates with genetic variation in alpha2-macroglobulin and tau: a population-based autopsy study. Ann Med.

[21] Coelho T, Maurer MS, Suhr OB (2013). THAOS - the Transthyretin Amyloidosis Outcomes Survey: initial report on clinical manifestations in patients with hereditary and wild-type transthyretin amyloidosis. Curr Med Res Opin.

[22] Merlini G, Westermark P (2004). The systemic amyloidoses: clearer understanding of the molecular mechanisms offers hope for more effective therapies. J Intern Med.

[23] Rowczenio DM, Noor I, Gillmore JD, Lachmann HJ, Whelan C, Hawkins PN, et al. Online registry for mutations in hereditary amyloidosis including nomenclature recommendations. Hum Mutat 2014;35. 10.1002/humu.22619 25044787

[24] Jacobson DR, Alexander AA, Tagoe C, Buxbaum JN (2015). Prevalence of the amyloidogenic transthyretin (TTR) V122I allele in 14 333 African-Americans. Amyloid.

[25] González-López E, Gagliardi C, Dominguez F, Quarta CC, de Haro-Del Moral FJ, Milandri A (2017). Clinical characteristics of wild-type transthyretin cardiac amyloidosis: disproving myths. Eur Heart J.

[26] Zadok OIB, Eisen A, Shapira Y, Monakier D, Iakobishvili Z, Schwartzenberg S, et al. Natural history and disease progression of early cardiac amyloidosis evaluated by echocardiography. Am J Cardiol 2020;133:126–33. 10.1016/j.amjcard.2020.07.050 32811652

[27] Chacko L, Martone R, Bandera F, Lane T, Martinez-Naharro A, Boldrini M (2020). Echocardiographic phenotype and prognosis in transthyretin cardiac amyloidosis. Eur Heart J.

[28] Bandera F, Martone R, Chacko L, Ganesananthan S, Gilbertson JA, Ponticos M, et al. Clinical importance of left atrial infiltration in cardiac transthyretin amyloidosis. JACC Cardiovasc Imaging; 2021; S1936–878X(21)00512-X. 10.1016/j.jcmg.2021.06.022 PMC8724534 34419399

[29] Riffel JH, Mereles D, Emami M, Korosoglou G, Kristen AV, Aurich M, et al. Prognostic significance of semiautomatic quantification of left ventricular long axis shortening in systemic light-chain amyloidosis. Amyloid 2015;22:45–53. 10.3109/13506129.2014.992515 25492308

[30] Cicco S, Solimando AG, Buono R, Susca N, Inglese G, Melaccio A (2020). Right heart changes impact on clinical phenotype of amyloid cardiac involvement: a single centre study. Life (Basel).

[31] Phelan D, Collier P, Thavendiranathan P, Popović ZB, Hanna M, Plana JC (2012). Relative apical sparing of longitudinal strain using two-dimensional speckle-tracking echocardiography is both sensitive and specific for the diagnosis of cardiac amyloidosis. Heart.

[32] Rapezzi C, Fontana M (2019). Relative left ventricular apical sparing of longitudinal strain in cardiac amyloidosis: is it just amyloid infiltration?. JACC Cardiovasc Imaging.

[33] Maceira AM, Joshi J, Prasad SK, Moon JC, Perugini E, Harding I (2005). Cardiovascular magnetic resonance in cardiac amyloidosis. Circulation.

[34] Zhao L, Tian Z, Fang Q. Diagnostic accuracy of cardiovascular magnetic resonance for patients with suspected cardiac amyloidosis: a systematic review and meta-analysis. BMC Cardiovasc Disord. 2016;16. 10.1186/s12872-016-0311-6 PMC4897958 27267362

[35] Fontana M, Pica S, Reant P, Abdel-Gadir A, Treibel TA, Banypersad SM (2015). Prognostic value of late gadolinium enhancement cardiovascular magnetic resonance in cardiac amyloidosis. Circulation.

[36] Karamitsos TD, Piechnik SK, Banypersad SM, Fontana M, Ntusi NB, Ferreira VM (2013). Noncontrast T1 mapping for the diagnosis of cardiac amyloidosis. JACC Cardiovasc Imaging.

[37] Fontana M, Banypersad SM, Treibel TA, Maestrini V, Sado DM, White SK (2014). Native T1 mapping in transthyretin amyloidosis. JACC Cardiovasc Imaging.

[38] Baggiano A, Boldrini M, Martinez-Naharro A, Kotecha T, Petrie A, Rezk T (2020). Noncontrast magnetic resonance for the diagnosis of cardiac amyloidosis. JACC Cardiovasc Imaging.

[39] Martinez-Naharro A, Treibel TA, Abdel-Gadir A, Bulluck H, Zumbo G, Knight DS (2017). Magnetic resonance in transthyretin cardiac amyloidosis. J Am Coll Cardiol.

[40] Knight DS, Zumbo G, Barcella W, Steeden JA, Muthurangu V, Martinez-Naharro A (2019). Cardiac structural and functional consequences of amyloid deposition by cardiac magnetic resonance and echocardiography and their prognostic roles. JACC Cardiovasc Imaging.

[41] Pan JA, Kerwin MJ, Salerno M (2020). Native T1 mapping, extracellular volume mapping, and late gadolinium enhancement in cardiac amyloidosis: a meta-analysis. JACC Cardiovasc Imaging.

[42] Chacko L, Boldrini M, Martone R, Law S, Martinez-Naharrro A, Hutt DF, et al. Cardiac magnetic resonance-derived extracellular volume mapping for the quantification of hepatic and splenic amyloid. Circ Cardiovasc Imaging. 2021 Apr 20:CIRCIMAGING121012506. 10.1161/CIRCIMAGING.121.012506 33876651

[43] Kotecha T, Martinez-Naharro A, Treibel TA, Francis R, Nordin S, Abdel-Gadir A (2018). Myocardial edema and prognosis in amyloidosis. J Am Coll Cardiol.

[44] Perugini E, Guidalotti PL, Salvi F, Cooke RMT, Pettinato C, Riva L (2005). Noninvasive etiologic diagnosis of cardiac amyloidosis using 99mTc-3,3-diphosphono-1,2-propanodicarboxylic acid scintigraphy. J Am Coll Cardiol.

[45] Hutt DF, Fontana M, Burniston M, Quigley AM, Petrie A, Ross JC (2017). Prognostic utility of the Perugini grading of 99mTc-DPD scintigraphy in transthyretin (ATTR) amyloidosis and its relationship with skeletal muscle and soft tissue amyloid. Eur Heart J Cardiovasc Imaging.

[46] Gillmore JD, Maurer MS, Falk RH, Merlini G, Damy T, Dispenzieri A (2016). Nonbiopsy diagnosis of cardiac transthyretin amyloidosis. Circulation.

[47] Scully PR, Morris E, Patel KP, Treibel TA, Burniston M, Klotz E (2020). DPD quantification in cardiac amyloidosis: a novel imaging biomarker. JACC Cardiovasc Imaging.

[48] Wollenweber T, Rettl R, Kretschmer-Chott E, Rasul S, Kulterer O, Rainer E (2020). In vivo quantification of myocardial amyloid deposits in patients with suspected transthyretin-related amyloidosis (ATTR). J Clin Med.

[49] Löfbacka V, Axelsson J, Pilebro B, Suhr OB, Lindqvist P, Sundström T (2021). Cardiac transthyretin amyloidosis ^99m^Tc-DPD SPECT correlates with strain echocardiography and biomarkers. Eur J Nucl Med Mol Imaging.

[50] Antoni G, Lubberink M, Estrada S, Axelsson J, Carlson K, Lindsjö L (2013). In vivo visualization of amyloid deposits in the heart with 11C-PIB and PET. J Nucl Med.

[51] Dorbala S, Vangala D, Semer J, Strader C, Bruyere JR, Di Carli MF (2014). Imaging cardiac amyloidosis: a pilot study using ^18^F-florbetapir positron emission tomography. Eur J Nucl Med Mol Imaging.

[52] Dietemann S, Nkoulou R (2019). Amyloid PET imaging in cardiac amyloidosis: a pilot study using ^18^F-flutemetamol positron emission tomography. Ann Nucl Med.

[53] Rosengren S, Skibsted Clemmensen T, Tolbod L, Granstam SO, Eiskjær H (2020). Diagnostic accuracy of [^11^C]PIB positron emission tomography for detection of cardiac amyloidosis. JACC Cardiovasc Imaging.

[54] Genovesi D, Vergaro G, Giorgetti A, Marzullo P, Scipioni M, Santarelli MF (2021). [18F]-Florbetaben PET/CT for differential diagnosis among cardiac immunoglobulin light chain, transthyretin amyloidosis, and mimicking conditions. JACC Cardiovasc Imaging.

[55] Kircher M, Ihne S, Brumberg J, Morbach C, Knop S, Kortüm KM (2019). Detection of cardiac amyloidosis with ^18^F-Florbetaben-PET/CT in comparison to echocardiography, cardiac MRI and DPD-scintigraphy. Eur J Nucl Med Mol Imaging.

[56] Lee SP, Suh HY, Park S, Oh S, Kwak SG, Kim HM (2020). Pittsburgh B compound positron emission tomography in patients with AL cardiac amyloidosis. J Am Coll Cardiol.

[57] Kim YJ, Ha S, Kim YI (2020). Cardiac amyloidosis imaging with amyloid positron emission tomography: a systematic review and meta-analysis. J Nucl Cardiol.

[58] Maurer MS, Schwartz JH, Gundapaneni B, Elliott PM, Merlini G, Waddington-Cruz M (2018). Tafamidis treatment for patients with transthyretin amyloid cardiomyopathy. N Engl J Med.

[59] Adams D, Gonzalez-Duarte A, O’Riordan WD, Yang C-C, Ueda M, Kristen A, et al. Patisiran, an RNAi therapeutic, for hereditary transthyretin amyloidosis. N Engl J Med. 2018;379:11–21. 10.1056/NEJMoa1716153 29972753

[60] Berk JL, Suhr OB, Obici L, Sekijima Y, Zeldenrust SR, Yamashita T (2013). Repurposing diflunisal for familial amyloid polyneuropathy: a randomized clinical trial. JAMA.

[61] Rosenblum H, Castano A, Alvarez J, Goldsmith J, Helmke S, Maurer MS. TTR (transthyretin) stabilizers are associated with improved survival in patients with TTR cardiac amyloidosis. Circ Heart Fail. 2018;11. 10.1161/CIRCHEARTFAILURE.117.004769 PMC5886729 29615436

[62] Koyama J, Minamisawa M, Sekijima Y, Ikeda S ichi, Kozuka A, Ebisawa S, et al. Left ventricular deformation and torsion assessed by speckle-tracking echocardiography in patients with mutated transthyretin-associated cardiac amyloidosis and the effect of diflunisal on myocardial function. Int J Cardiol Heart Vasc. 2015;9:1–10. 10.1016/j.ijcha.2015.07.010 PMC5497336 28785698

[63] Lohrmann G, Pipilas A, Mussinelli R, Gopal DM, Berk JL, Connors LH (2020). Stabilization of cardiac function with diflunisal in transthyretin (ATTR) cardiac amyloidosis. J Card Fail.

[64] Shintani Y, Okada A, Morita Y, Hamatani Y, Amano M, Takahama H, et al. Monitoring treatment response to tafamidis by serial native T1 and extracellular volume in transthyretin amyloid cardiomyopathy. ESC Heart Failure. 2019;6:232. 10.1002/ehf2.12382 PMC6352892 30478886

[65] Solomon SD, Adams D, Kristen A, Grogan M, González-Duarte A, Maurer MS (2019). Effects of patisiran, an RNA interference therapeutic, on cardiac parameters in patients with hereditary transthyretin-mediated amyloidosis: analysis of the APOLLO study. Circulation.

[66] Fontana M, Martinez-Naharro A, Chacko L, Rowczenio D, Gilbertson JA, Whelan CJ (2021). Reduction in CMR derived extracellular volume with patisiran indicates cardiac amyloid regression. JACC Cardiovasc Imaging.

[67] Benson MD, Waddington-Cruz M, Berk JL, Polydefkis M, Dyck PJ, Wang AK (2018). Inotersen treatment for patients with hereditary transthyretin amyloidosis. N Engl J Med.

[68] Benson MD, Dasgupta NR, Rissing SM, Smith J, Feigenbaum H (2017). Safety and efficacy of a TTR specific antisense oligonucleotide in patients with transthyretin amyloid cardiomyopathy. Amyloid.

[69] Dasgupta NR, Rissing SM, Smith J, Jung J, Benson MD (2020). Inotersen therapy of transthyretin amyloid cardiomyopathy. Amyloid.

[70] Efficacy and safety of AG10 in subjects with transthyretin amyloid cardiomyopathy - Full Text View - ClinicalTrials.gov [Internet]. 2019 [cited 2021 Nov 20]. Available from: https://clinicaltrials.gov/ct2/show/NCT03860935

[71] HELIOS-B: a study to evaluate vutrisiran in patients with transthyretin amyloidosis with cardiomyopathy - Full Text View - ClinicalTrials.gov [Internet]. 2019 [cited 2021 Nov 20]. Available from: https://clinicaltrials.gov/ct2/show/NCT04153149

[72] A study of PRX004 in subjects with amyloid transthyretin (ATTR) amyloidosis - Full Text View - ClinicalTrials.gov [Internet]. 2017 [cited 2021 Nov 20]. Available from: https://clinicaltrials.gov/ct2/show/NCT03336580

[73] Study to evaluate safety, tolerability, pharmacokinetics, and pharmacodynamics of NTLA-2001 in patients with hereditary transthyretin amyloidosis with polyneuropathy (ATTRv-PN) - Full Text View - ClinicalTrials.gov [Internet]. 2020 [cited 2021 Nov 20]. Available from: https://clinicaltrials.gov/ct2/show/NCT04601051

[74] Gillmore JD, Gane E, Taubel J, Kao J, Fontana M (2021). CRISPR-Cas9 in vivo gene editing for transthyretin amyloidosis. N Engl J Med.

[75] A study of daratumumab monotherapy in previously untreated patients with stage 3B light chain (AL) amyloidosis – Full Text View – ClinicalTrials.gov [Internet]. 2021 [cited 2021 Nov 20]. Available from: https://clinicaltrials.gov/ct2/show/NCT04131309

[76] Tuzovic M, Kobayashi Y, Wheeler M, Barrett C, Liedtke M, Lafayette R (2017). Functional cardiac recovery and hematologic response to chemotherapy in patients with light-chain amyloidosis (from the Stanford University Amyloidosis Registry). Am J Cardiol.

[77] Salinaro F, Meier-Ewert HK, Miller EJ, Pandey S, Sanchorawala V, Berk JL (2017). Longitudinal systolic strain, cardiac function improvement, and survival following treatment of light-chain (AL) cardiac amyloidosis. Eur Heart J Cardiovasc Imaging.

[78] •• Cohen OC, Ismael A, Pawarova B, Manwani R, Ravichandran S, Law S, et al. Longitudinal strain is an independent predictor of survival and response to therapy in patients with systemic AL amyloidosis. Eur Heart J. 2021:ehab507. **This study provides evidence of longitudinal strain being an important predictor of survival that improves in response to treatment in patients with AL-CA.** 10.1093/eurheartj/ehab507 34472567

[79] Meier-Ewert HK, Sanchorawala V, Berk J, Finn KT, Skinner M, Seldin DC (2011). Regression of cardiac wall thickness following chemotherapy and stem cell transplantation for light chain (AL) amyloidosis. Amyloid.

[80] Madan S, Kumar SK, Dispenzieri A, Lacy MQ, Hayman SR, Buadi FK (2012). High-dose melphalan and peripheral blood stem cell transplantation for light-chain amyloidosis with cardiac involvement. Blood.

[81] Pun SC, Landau HJ, Riedel ER, Jordan J, Yu AF, Hassoun H (2018). Prognostic and added value of 2D global longitudinal strain for prediction of survival in patients with light chain (AL) amyloidosis undergoing autologous hematopoietic cell transplant. J Am Soc Echocardiogr.

[82] Hwang IC, Koh Y, Park JB, Yoon YE, Kim HL, Kim HK (2021). Time trajectory of cardiac function and its relation with survival in patients with light-chain cardiac amyloidosis. Eur Heart J.

[83] • Martinez-Naharro A, Abdel-Gadir A, Treibel TA, Zumbo G, Knight DS, Rosmini S, et al. CMR-verified regression of cardiac AL amyloid after chemotherapy. JACC Cardiovasc Imaging; 2018;11:152–4. **This study provides evidence of CMR-derived ECV regression in response to chemotherapy in patients with AL-CA.** 10.1016/j.jcmg.2017.02.012 28412427

[84] Patel RK, Fontana M, Ruberg FL (2021). Cardiac amyloidosis: multimodal imaging of disease activity and response to treatment. Circ Cardiovasc Imaging.

[85] Lane T, Fontana M, Martinez-Naharro A, Quarta CC, Whelan CJ, Petrie A (2019). Natural history, quality of life, and outcome in cardiac transthyretin amyloidosis. Circulation.

[86] de Marvao A, Dawes TJW, Howard JP, O’Regan DP (2020). Artificial intelligence and the cardiologist: what you need to know for 2020. Heart.

[87] Howard JP, Francis DP. Machine learning with convolutional neural networks for clinical cardiologists. Heart; 2021; :heartjnl-2020–318686. 10.1136/heartjnl-2020-318686 34301771

[88] Dey D, Slomka PJ, Leeson P, Comaniciu D, Shrestha S, Sengupta PP (2019). Artificial intelligence in cardiovascular imaging: JACC state-of-the-art review. J Am Coll Cardiol.

[89] Karagodin I, Carvalho Singulane C, Woodward GM, Xie M, Tucay ES, Tude Rodrigues AC (2021). Echocardiographic correlates of in-hospital death in patients with acute COVID-19 infection: the World Alliance Societies of Echocardiography (WASE-COVID) study. J Am Soc Echocardiogr.

